# Understanding of Pharmacy Students towards Antibiotic Use, Antibiotic Resistance and Antibiotic Stewardship Programs: A Cross-Sectional Study from Punjab, Pakistan

**DOI:** 10.3390/antibiotics10010066

**Published:** 2021-01-12

**Authors:** Khezar Hayat, Shazia Jamshed, Meagen Rosenthal, Noman Ul Haq, Jie Chang, Muhammad Fawad Rasool, Usman Rashid Malik, Anees Ur Rehman, Kashif Maqbool Khan, Yu Fang

**Affiliations:** 1 Department of Pharmacy Administration and Clinical Pharmacy, School of Pharmacy, Xi’an Jiaotong University, Xi’an 710061, China; khezar.hayat@uvas.edu.pk (K.H.); jiechang@xjtu.edu.cn (J.C.); usmanmalik_ucp@hotmail.com (U.R.M.); 2 Center for Drug Safety and Policy Research, Xi’an Jiaotong University, Xi’an 710061, China; 3 Shaanxi Centre for Health Reform and Development Research, Xi’an 710061, China; 4 Institute of Pharmaceutical Sciences, University of Veterinary and Animal Sciences, Lahore 54000, Pakistan; Kashif.maqbool@uvas.edu.pk; 5 Department of Clinical Pharmacy and Practice, Faculty of Pharmacy, Universiti Sultan Zainal Abidin, Terengganu 22200, Malaysia; shaziajamshed@unisza.edu.my; 6 Qualitative Research-Methodological Application in Health Sciences Research Group, Kulliyyah of Pharmacy, International Islamic University Malaysia (IIUM), Kuantan 25200, Malaysia; 7 Department of Pharmacy Administration, School of Pharmacy, University of Mississippi, Oxford, MS 38677, USA; mmrosent@olemiss.edu; 8 Department of Pharmacy Practice, Faculty of Pharmacy, University of Balochistan, Quetta 87900, Pakistan; nomanhaq79@gmail.com; 9 Department of Pharmacy Practice, Faculty of Pharmacy, Bahauddin Zakariya University, Multan 60800, Pakistan; fawadrasool@bzu.edu.pk (M.F.R.); aneesurrehmanr90@gmail.com (A.U.R.)

**Keywords:** antibiotic use, antibiotic resistance, stewardship programs, pharmacy students, knowledge

## Abstract

Antibiotic resistance (ABR) is a significant issue for public health globally. An adequate understanding of ABR and the approaches used to tackle ABR, including antibiotic stewardship programs, are vital. This study aimed to get an insight into antibiotic use, ABR, and antibiotic stewardship programs among pharmacy students of Punjab, Pakistan. This multicenter study was undertaken among final (fifth) year undergraduate pharmacy students of 7 universities of Punjab, Pakistan. A paper-based self-administered questionnaire comprising 48-items was utilized for data collection. Descriptive and inferential statistics were employed for data analysis. This study included a total of 296 respondents with a response rate of 85.8%. Most of the students had an average understanding of antibiotic use (59.8%), ABR (42.6%), ABR mechanisms (48.0%), and factors of ABR (51.7%). Only 21.6% of students have heard about antibiotic stewardship programs. More than half of the students believed that educating and training healthcare professionals (53.4%) and medical students (57.8%) about the prescribing and judicial usage of antibiotics could reduce the ABR burden. The awareness of most of the pharmacy students about certain aspects of antibiotic use, ABR, and stewardship programs was suboptimal.

## 1. Introduction

Antibiotics are used to treat multiple infections due to their inherited ability to restrict the growth of or killing pathogenic microorganisms [1]. However, the continuous irrational, and injudicious use of antibiotics has led to antibiotic resistance (ABR). ABR is a silent tsunami affecting every part of the globe [2]. It has significant implications for healthcare spending due to the resulting increase in morbidity and mortality. Every year, nearly 2 million people are affected by resistant infections causing the death of 23,000 patients with a loss of 55 billion USD in the United States (US) [3]. Likewise, 25,000 deaths and 1.5 billion euros are lost in Europe each year [4]. The catastrophic effects attributed to ABR are continuously increasing due to the unavailability of new antibiotics.

The injudicious use of antibiotics in community and hospital settings is a significant driver to increase the ABR problem globally. Studies have shown that 20% to 50% of antibiotics prescribed in hospitals are inappropriate [3]. Similarly, more than half of the antibiotics dispensed from community pharmacies are irrational [5]. The situation of ABR in developing countries is worse owing to the climbing incidence of infectious diseases, lack of adequate knowledge among healthcare professionals, insufficient training, inadequate diagnostic facilities, lack of standard treatment guidelines, and antibiotic sale without prescription [6,7].

In this context, adequate knowledge of healthcare professionals about rational antibiotic use and antimicrobial resistance (AMR) prevention could play a significant role in limiting ABR momentum and sustaining the effectiveness of antibiotics [8,9]. One of the most integral elements of the World Health Organization (WHO) global action plan to manage ABR is to enhance understanding and awareness about antibiotics among healthcare professionals and the public by effective education, training, and communication [10]. The education and training about appropriate antibiotic use during undergraduate education have a positive impact on the attitude and behavior of healthcare professionals regarding the use of antibiotics [11]. Thus, this training is paramount for doctors, pharmacists, and nurses.

The role of pharmacists in medicine use, including managing ABR through antibiotic stewardship programs (programs that work in collaboration with healthcare professionals aiming to improve patient outcomes by minimizing antibiotic resistance, therapy cost, and risk of resistant infections), is manifested in previously published research [12,13,14]. Education and adequate pharmacists’ training could modify the behavior of doctors, nurses, and consumers as they are the most accessible professionals in the community [13,15]. The professional practice of pharmacists could become substandard in some developing countries, including Pakistan, China, and India if they have insufficient training and education; as a consequence, pharmacists may recommend and supply antibiotics inappropriately [16,17]. Pharmacists with comprehensive education and training on AMR and stewardship programs could play a leading role in changing the community’s behavior about antibiotic use and ABR [18].

Pakistan is a developing country where the supply of antibiotics is regulated by well-established legislation, and antibiotics can only be dispensed with a valid prescription written by a medical practitioner [19]. A pharmacist needs to complete a five-year Pharm D (Doctor of Pharmacy) program before practicing [20]. It is expected that pharmacy students will be well trained to address the health-related issues of the public. However, little is known about pharmacy students’ understanding of antibiotic use, antibiotic resistance, and antibiotic stewardship programs in developing countries such as Pakistan. Therefore, this study was designed to investigate the understanding of the fifth (final) year pharmacy students in seven universities in Pakistan about antibiotic use, antibiotic resistance, and antibiotic stewardship programs.

## 2. Methodology

### 2.1. Study Setting

This cross-sectional study was conducted in seven universities (three public and four private) in the Punjab province of Pakistan from October 2018 to January 2019. A self-administered questionnaire was utilized for data collection among respondents. The eligibility criteria of this study included students who provided written informed consent, were willing to participate, and were enrolled in the final (fifth) year Pharm D. Students who were enrolled in other disciplines instead of pharmacy or junior Pharm D students were excluded.

### 2.2. Questionnaire Development

The questionnaire used for this survey was developed from a thorough literature survey [21,22,23,24,25]. The validity of the questionnaire was established by 2 professors of pharmacy background and 10 undergraduate pharmacy students. Minor changes to the final version of the questionnaire were made as per the recommendations of the experts.

There were seven sections of the questionnaire with 48-items (supplementary file). The demographic information such as age, gender, and type of university was obtained in the first section. In the second section, 14 questions were asked to determine pharmacy students’ understanding of antibiotic use. The third section had questions related to antibiotic resistance and antibiotic stewardship programs. The fourth section focused on pharmacy students’ understanding of the mechanism of antibiotic resistance with six questions. In the fifth section, information about factors affecting antibiotic resistance was recorded. Three options, such as “yes”, “no”, and “do not know”, were provided as answer options from sections 1 to 6. In the last section, the attitude of students about strategies to reduce antibiotic resistance was measured on a 5-point Likert scale measured from “strongly agree” to “strongly disagree.” Each correct response in sections two to five was given one mark, and zero marks were given to each incorrect/do not know response. The overall score of each section was grouped into three main categories: poor (score 1–5 for antibiotic use section, score 1–3 for antibiotic resistance section, and 1–2 for the mode of ABR and factors of ABR section), average (score 6–10 for antibiotic use section, score 4–6 for antibiotic resistance section, 3–4 for the mode of ABR and factors of ABR section), and good (score >10 for antibiotic use section, score >6 for antibiotic resistance section, >4 for the mode of ABR and factors of ABR section).

A pilot study was executed with 20 final year pharmacy students. Cronbach-alpha test was used to determine the reliability and internal consistency of the questionnaire. The value of the reliability coefficient was 0.75, which was in an acceptable range. The pilot sample was excluded in the final sample of the research.

### 2.3. Sample Size

The sample size (*n* = 227) was calculated using an online sample size software (Raosoft) with a 5% margin of error, 95% confidence interval, and 50% response distribution.

### 2.4. Data Collection

The data were collected by data collectors (undergraduate pharmacy students) trained by the study investigator about the study aims, questionnaire administration, and checking of the questionnaire’s completeness. They approached a cohort of final year pharmacy students and distributed the questionnaire. It took 15–20 min to complete the survey. Any query raised by students was adequately addressed by the data collectors.

### 2.5. Ethics Approval

This study was conducted by following the Declaration of Helsinki. Participants were briefed about the objectives of the study, volunteer participation, and the right to withdraw. All participants provided informed consent prior to the study. Ethics approval was also obtained from Xi’an Jiaotong University (Ref: Phar-2018-015).

### 2.6. Statistical Analysis

Descriptive statistics were used to present data in percentages and frequencies. The normality of the data was assessed by Kolmogorov–Smirnov and Shapiro–Wilk tests. Median and interquartile ranges (IQRs) were measured as the data distribution was non-normal. Kruskal–Wallis and Mann–Whitney tests were computed on continuous data. Median antibiotic use score (measuring understanding about antibiotic use), median knowledge score (measuring knowledge about antibiotic resistance), median ABR mechanism score (measuring understanding about resistance mechanisms of antibiotics), median ABR factors score (measuring factors of antibiotic resistance), and median attitude score (measuring attitude towards approaches used to reduce antibiotic resistance) were also calculated and compared with demographics such as age, gender, and type of university. All data were analyzed using the Statistical Package for the Social Sciences (SPSS Inc, version 18, IBM, Chicago, IL, USA) with *p* < 0.05 as statistically significant.

## 3. Results

### 3.1. Demographics

A total of 345 respondents were approached in seven universities of Punjab, Pakistan, and 296 respondents participated in this study with a response rate of 85.8%. Most of the respondents were female (*n* = 184, 62.2%) and had age in between 22–24 years (*n* = 231, 78.0%). The respondents’ participation from public and private universities was the same (public = 148, 50% vs. private = 148, 50%) as indicated in Table 1.

### 3.2. Understanding towards Antibiotic Use

**Figure 1:**
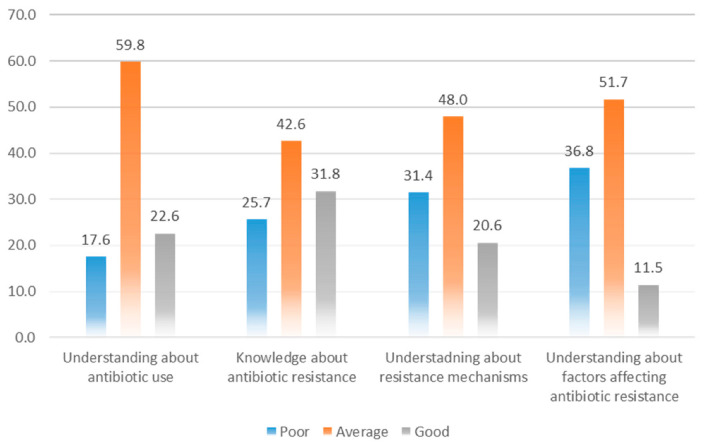
Understanding of respondents towards various aspects of antibiotic use and antibiotic resistance.

Overall, 177 (59.9%) of the students fell into the average understanding category, whereas only 67 (22.6%) respondents had a good understanding (Figure 1). Nevertheless, most of the respondents agreed that antibiotics are frequently prescribed antibacterials in both public (*n* = 261, 88.2%) and private healthcare settings (*n* = 219, 74.0%). They were aware that antibiotics should not be left over at home for future use (*n* = 211, 71.3%), and patients should complete the antibiotic course even when symptoms get improved (*n* = 170, 57.4%). The understanding of the respondents was limited in certain aspects of antibiotic use such as more than half of the respondents (*n* = 152, 51.4%) said that antibiotics should be given as a preventive medication to tackle future infections, and 149 (50.3%) respondents were unable to identify that diphenhydramine is not an antibiotic as shown in Table 2. The median antibiotic use score was significantly higher among respondents of public universities than private (Median = 1.5, IQR = 1.00, vs. Median = 1.0, IQR = 0.88; *p* < 0.001).

### 3.3. Understanding of Antibiotic Resistance

**Figure 2:**
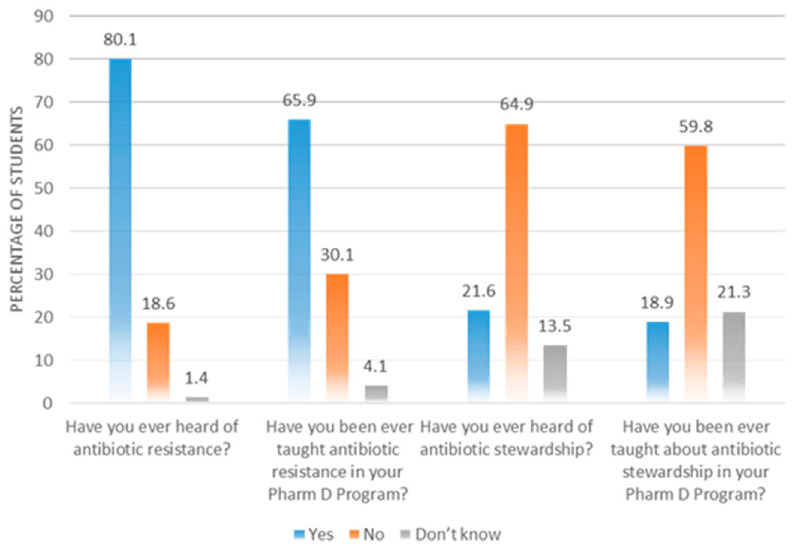
Awareness of students about antibiotic resistance and stewardship.

The majority of the students (*n* = 237, 80.1%) were aware of the term “antibiotic resistance” as this was taught to them during their Pharm D (*n* = 195, 65.9%). However, the respondents were not familiar with the term “antibiotic stewardship program” (*n* = 192, 64.9%), and 177 (59.8%) respondents said that they had not been taught about antibiotic stewardship (Figure 2).

A large number of respondents (*n* = 126, 42.6%) had an average level of ABR related knowledge, whereas only 94 (31.8%) had good knowledge (Figure 1). The respondents rightly said that inadequate therapy (*n* = 189, 63.9%) and doses could lead to ABR (*n* = 222, 75.0%). Likewise, 160 (54.1%) respondents were aware that healthcare professionals could transfer-resistant strains from an infected patient to a healthy person. A detailed understanding of ABR is listed in Table 3.

The respondents aged 19–21 years had significantly higher understanding of ABR compared to those with age >24 years (Median = 1.5, IQR = 1.00, vs. Median = 1.0, IQR = 1.00; *p* < 0.001). Similarly, the median score of respondents of public universities was noted to be higher than respondents of private universities (Median = 1.5, IQR = 1.00, vs. Median = 1.0, IQR = 0.00; *p* < 0.001) as indicated in Table 7.

### 3.4. Understanding of the Mode of Action of Antibiotics

The understanding of most of the respondents (*n* = 142, 48%) about the resistance mechanism was average; however, 61 (20.6%) had a good understanding (Figure 1). Many students agreed that beta-lactamases cause the breakdown of beta-lactam antibiotics (*n* = 213, 72.0%), and bacteria could use drug efflux mechanisms to produce ABR (*n* = 164, 55.4%) as shown in Table 4. However, a minimal number of respondents answered correctly about statements such as “There is no resistance to penicillin for Streptococcus pyogenes” (*n* = 90, 30.4%). The respondents with age 19–21 years were more aware of the ABR mechanisms than 22–24 years (Median = 2.0, IQR = 0.50, Median = 1.5, IQR = 1.00; *p* = 0.002) (Table 7).

### 3.5. Understanding of the Factors of Antibiotic Resistance

More than half of the respondents (*n* = 153, 51.7%) had an average understanding of ABR factors (Figure 1). The respondents knew that mutation is a contributing factor leading to protein change in bacteria (*n* = 188, 63.5%), and broad-spectrum antibiotics used as first-line therapy for minor infections could amplify ABR risk (*n* = 189, 63.9%). A total of 194 (65.5%) respondents believed that healthcare-associated infections contribute to ABR. Alternatively, 87 (29.4) agreed that commercially available biocide antiseptics in soaps are not useful for patients with skin infections (Table 5). The understanding of students from public universities was found to be significantly higher about the factors of ABR compared to private university students (Median = 1.0, IQR = 1.00 vs. Median = 1.0, IQR = 0.00; *p* < 0.001).

### 3.6. Attitude toward Reducing Antibiotic Resistance

The attitude of a large number of respondents was positive towards reducing the ABR burden. More than half of the respondents strongly agreed that educating healthcare professionals (*n* = 158, 53.4%) and medical students about the prescribing and usage of antibiotics could reduce the ABR burden (*n* = 150, 50.7%). A total of 171 (57.8%) respondents perceived that the rational use of antibiotics could reduce the hospital’s expense. Likewise, 155 (52.4%) said that antibiotic prescribing via phone is not suitable for patients. Most of the respondents agreed that patients should be advised to participate in antibiotic awareness campaigns to enhance their knowledge. 123 (41.6%) of the respondents believed that pharmacists should dispense antibiotics according to the demand of patients (Table 6). The median attitude score of respondents was also significantly associated with gender, age, and university type (Table 7).

## 4. Discussion

This is the first comprehensive study that illustrates the understanding of final-year pharmacy students from 7 Pakistani universities about antibiotic use, ABR, and antibiotics stewardship programs. The findings of this study show that students have an average understanding of certain aspects of antibiotic use and ABR, and their attitude is positive towards the approaches used to limit progression in ABR.

Most of the respondents of our study believed that antibiotics are widely used antimicrobials in public and private hospitals. Similar results were reported in a Malaysian study [21]. Additionally, a recent study described that half of the antibiotics used in hospitalized patients are inappropriate [26].

Nearly half of our survey respondents (46.6%) agreed that influenza could be treated with antibiotics. The current research is in concordance with a study conducted in Saudi-Arabia, which reported that more than one-fourth of respondents (medical students) wrongly perceived that antibiotics are useful against viral infections [27]. Unfortunately, the use of antibiotics in viral diseases such as influenza is widespread in the community and hospitals [28], which could be due to a lack of optimum antibiotic-related knowledge among healthcare workers and the public [29,30].

A total of 74.7% of the respondents said that antibiotic dispensing does not require a prescription in Pakistan. According to the Drug Act 1967 of Pakistan, dispensing antibiotics without a valid prescription is prohibited; however, the irrational dispensing of antibiotics is widespread [6,31]. Many studies from Pakistan have reported that pharmacists and pharmacy technicians routinely dispense antibiotics without inquiring about a prescription [32,33,34]. The respondents of our study righty pointed out that non-compliance to regulations often permits the purchase of antibiotics without a prescription from community pharmacies. The government should take strict measures against irrational antibiotic dispensing, and appropriate punishments should be given for non-compliance with regulations. Besides, the government should advise community pharmacies and other drug outlets to maintain a record of the sale of antibiotics coupled with photocopies of the prescriptions.

More than half of the survey respondents agreed that healthcare workers could act as a vector to transfer resistant strains of pathogens within a hospital. This complies with various studies [35,36], such as a study conducted in Tanzania that found that healthcare workers, including nurses, had a high percentage of MRSA carriage [37]. Like a previous study [21], many respondents believed that switching empiric therapy to definitive therapy in the presence of culture and sensitivity tests could help reduce ABR. Nearly two-thirds of the respondents understood that inadequate antibiotic therapy duration could lead to ABR, as found in a study conducted in Malaysian pharmacy students [21]. It is recommended that antibiotics should be used for an optimal duration as recommended by healthcare professionals, and their inappropriate use could potentiate the risk of ABR [38,39].

Only 30.4% of our study respondents correctly answered that there is no resistance of *Streptococcus pyogenes* against penicillin. However, this percentage is still higher than a study conducted on physicians, where only 21% of physicians were able to show this knowledge [40]. On the other hand, this correctness is far less than a Malaysian study (56.6%) [21].

Most of our study respondents were familiar with ABR as this was taught to them during their PharmD; however, their awareness towards antibiotic stewardship programs was limited. Our previous study conducted with clinicians also showed limited awareness of stewardship programs [41]. Although ABR is now a part of the curriculum in many pharmacy schools, information about stewardship programs is still missing. Many countries, such as South Africa and the United States, have successfully included antibiotic stewardship programs in their pharmacy curricula [42,43].

Surprisingly, 61.5% of students advocated using soaps containing biocide antiseptics for patients with cutaneous infections. This percentage is far more than a previous study [21]. This wrong perception among students might come from the fact that antibiotic creams are often prescribed to treat skin infections.

A large number of students were in agreement that healthcare professionals and medical students should have adequate education and training regarding optimum antibiotic prescribing and ABR. Many previous studies have highlighted the importance of education and training of healthcare professionals and students [44,45]. Besides, studies have shown the positive impact of education on antibiotic prescribing practices of healthcare professionals [46]. A considerable number of students thought that antibiotics should be provided to meet the demands of patients. This negative attitude may be due to the self-medication trend among medical students. This belief can be further motivated by the urge to get prompt relief from illness and avoid clinician fees.

In this study, the understanding of public sector university students about various ABR aspects was significantly higher than that of private universities. This may be because pharmacy institutes in public universities of Pakistan are equipped with an adequate number of trained and experienced academic staff coupled with sufficient laboratory facilities.

A better theoretical understanding of antibiotics is integral for a better practical attitude of pharmacy students. There is a need to revise and continuously update the Pharm D curriculum by adding comprehensive information about antibiotic resistance and antibiotic stewardship programs [47,48]. In addition, the pharmacy students should be provided with an opportunity to participate in hospital ward rounds to better understand the concept of ABR. Furthermore, the government should implement antibiotic stewardship in all health care settings of Pakistan to provide a discussion forum for medical doctors and pharmacy students to get solutions to ABR based on evidence [6,33,49].

The authors acknowledge certain limitations within this study. First, this study was conducted in selected universities, opting for a convenience sampling technique, which may cause selection bias. Second, the study was undertaken in only one province of Pakistan, and therefore, the results are unable to offer generalizability. Nevertheless, this is an exploratory study that provides the latest insight of final-year pharmacy students about ABR and antibiotic stewardship programs.

## 5. Conclusions

This study undertaken with final-year pharmacy students from Pakistani universities has highlighted gaps in understanding the main aspects of antibiotic use, ABR, and stewardship programs. However, students showed positive attitudes towards different approaches used to manage ABR in Pakistan. A significant association between the type of university with antibiotic use, ABR, factors of ABR and attitude towards ABR eradication were noted. Interventions such as revising the pharmacy curricula by incorporating courses related to ABR and stewardship, coupled with adequate problem-based practical training of pharmacy students are recommended. Future studies covering a larger proportion of pharmacy students across the country are needed to confirm the findings of this study.

## Figures and Tables

**Table 1:** Demographic characteristics of students (*n* = 296).

Variable	Frequency (*n*)	Percentage (%)
**Gender**		
Male	112	37.8
Female	184	62.2
**Age (years)**		
19–21	59	19.9
22–24	231	78.0
>24	6	2.0
**Type of University**		
Public	148	50.0
Private	148	50.0

**Table 2:** Understanding about antibiotic use *n* (%).

Questions	Yes	No	Do Not Know	Correct Rate	Median (IQR)
Antibiotics are the most commonly prescribed anti-infective agents by public health-care sector facilities	261 (88.2)	30 (10.1)	5 (1.7)	261 (88.2)	1 (0)
Antibiotics are the most commonly prescribed anti-infective agents by private health-care sector facilities	219 (74.0)	67 (22.6)	10 (3.4)	219 (74.0)	1 (1)
Common cold if treated with antibiotics will make the patients recover more quickly	129 (43.6)	130 (43.9)	37 (12.5)	130 (43.9)	2 (1)
Antibiotics should be prescribed as preventive measures to fight against future microbial attacks	152 (51.4)	124 (41.9)	20 (6.8)	124 (41.9)	1 (1)
Antibiotics cannot treat influenza	134 (45.3)	138 (46.6)	24 (8.1)	134 (45.3)	2 (1)
Antibiotics are indicated to relieve pain	100 (33.8)	164 (55.4)	32 (10.8)	164 (55.4)	2 (1)
Antibiotics might develop allergy in susceptible individuals	215 (72.6)	58 (19.6)	23 (7.8)	215 (72.6)	1 (1)
Diphenhydramine is an antibiotic used in treating upper respiratory infections	149 (50.3)	117 (39.5)	30 (10.1)	117 (39.5)	1 (1)
Cefotaxime belongs to the third-generation cephalosporins	179 (60.5)	69 (23.3)	48 (16.2)	179 (60.5)	1 (1)
Patients can stop taking antibiotics when the symptoms are improving	98 (33.1)	161 (54.4)	37 (12.5)	161 (54.4)	2 (1)
Keeping the left-over antibiotic course for the next time treatment of the same type of infection is a good practice	68 (23.0)	211 (71.3)	17 (5.7)	211 (71.3)	2 (0)
Antibiotics treatment can eliminate most of the sensitive bacterial cells from patients	170 (57.4)	100 (33.8)	26 (8.8)	170 (57.4)	1 (1)
Antibiotics can be obtained without a prescription in Pakistan	221 (74.7)	63 (21.3)	12 (4.1)	221 (74.7)	1 (1)
Antibiotics are the first line of treatment in sore throat	123 (41.6)	132 (44.6)	41 (13.9)	132 (44.6)	2 (1)

**Table 3:** Understanding about antibiotic resistance use *n* (%).

Questions	Yes	No	Do Not Know	Correct Rate	Median (IQR)
A resistant bacterium cannot spread in healthcare institutions	121 (40.9)	168 (56.8)	7 (2.4)	168 (56.8)	2 (1)
Health care workers serve as vectors carrying resistant strains from infected patients to normal patients	160 (54.1)	120 (40.5)	16 (5.4)	160 (54.1)	1 (1)
Exposure to antibiotics appears to be the principal risk factor for the emergence of antibiotic-resistant bacteria	215 (72.6)	59 (19.9)	22 (7.4)	215 (72.6)	1 (1)
Inadequate duration of therapy contributes to antibiotic resistance leading to poor patient compliance	189 (63.9)	90 (30.4)	17 (5.7)	189 (63.9)	1 (1)
Inadequate doses contribute to antibiotic resistance due to poorly designed dosing regimen	222 (75.0)	57 (19.3)	17 (5.7)	222 (75.0)	1 (0.75)
Antimicrobial resistance can be minimized through changing empiric therapy to a selected narrow-spectrum therapy in response to the availability of culture and sensitivity results	219 (74.0)	60 (20.3)	17 (5.7)	219 (74.0)	1 (1)
Cross-resistance is the condition in which resistance occurs to a particular antibiotic that often results in resistance to other antibiotics, usually from a similar class	185 (62.5)	83 (28.0)	28 (9.5)	185 (62.5)	1 (1)
Lack of enforcement regulation sometimes permits antibiotics to be purchased without a prescription from pharmacies	206 (69.6)	80 (27.0)	10 (3.4)	206 (69.6)	1 (1)

**Table 4:** Understanding of respondents about the resistance mechanisms of antibiotics *n* (%).

Questions	Yes	No	Do Not Know	Correct Rate	Median (IQR)
Beta-lactamase is an enzyme produced by bacteria that breakdown the beta-lactam antibiotics	213 (72.0)	72 (24.3)	11 (3.7)	213 (72.0)	1 (1)
Bacteria acquire efflux pumps that extrude the antibacterial agent from the cell before it can reach its target site and exert its effect	164 (55.4)	67 (22.6)	65 (22.0)	164 (55.4)	1 (1)
There is no resistance to penicillin for *Streptococcus pyogenes* bacteria	90 (30.4)	113 (38.2)	93 (31.4)	90 (30.4)	2 (2)
Bacteriostatic antibiotics are the same as bactericidal antibiotics	48 (16.2)	191 (64.5)	57 (19.3)	191 (64.5)	2 (0)
Antibiotic refers to any agent used to kill or inhibit the growth of microorganisms	180 (60.8)	70 (23.6)	46 (15.5)	180 (60.8)	1 (1)
Enterococcus is a vancomycin-resistant bacterium	115 (38.9)	79 (26.7)	102 (34.5)	115 (38.9)	2 (2)

**Table 5:** Understanding about factors contributing to antibiotic use *n* (%).

Questions	Yes	No	Do Not Know	Correct Rate	Median (IQR)
The mutation is a prevalence factor in changing the bacterial protein, which is often the target of antibiotic treatment	188 (63.5)	61 (20.6)	47 (15.9)	188 (63.5)	1 (1)
The use of broad-spectrum antibiotics (e.g., 4th generation cephalosporins) as initial therapy for mild infection may increase the risk of antibiotic resistance	189 (63.9)	83 (28.0)	24 (8.1)	189 (63.9)	1 (1)
The use of commercially available biocide antiseptics in soaps is highly recommended to patients who have skin infections	182 (61.5)	87 (29.4)	27 (9.1)	87 (29.4)	1 (1)
Antibiotic resistance tends to be a feature of urban social change	199 (67.2)	58 (19.6)	39 (13.2)	199 (67.2)	1 (1)
Healthcare acquired infections are a breeding ground for antimicrobial resistance	194 (65.5)	65 (22.0)	37 (12.5)	194 (65.5)	1 (1)

**Table 6:** Attitude towards minimizing antibiotic resistance *n* (%).

Questions	Strongly Agree	Agree	Neutral	Disagree	Strongly Disagree	Median (IQR)
Educating healthcare professional in terms of appropriate antibiotic prescribing	158 (53.4)	99 (33.4)	31 (10.5)	4 (1.4)	4 (1.4)	1 (1)
Formal teaching on the proper usage of antimicrobial agents among health care students may minimize the phenomenon of antibiotic resistance	150 (50.7)	101 (34.1)	27 (9.1)	17 (5.7)	1 (0.3)	1 (1)
Antimicrobial education is needed to be well received by healthcare practitioners	164 (55.4)	91 (30.7)	23 (7.8)	12 (4.1)	6 (2.0)	1 (1)
The usage of antibiotics must be related to specialities to enhance the awareness of antibiotic resistance	126 (42.6)	132 (44.6)	17 (5.7)	13 (4.4)	8 (2.7)	2 (1)
Appropriate use of the antibiotic may not have any impact on the hospital’s total cost expenses on medications	18 (6.1)	0(0.0)	49 (16.6)	171 (57.8)	58 (19.6)	4 (0)
Prescribing antibiotics over the phone is a good patient care	67 (22.6)	0(0.0)	58 (19.6)	155 (52.4)	16 (5.4)	4 (1)
The patient should be advised to keep part of the antibiotic course for another occasion which will help them in cutting down the medical cost	21 (7.1)	116 (39.2)	56 (18.9)	60 (20.3)	43 (14.5)	3 (2)
Pharmacists should be encouraged to dispense antibiotics to meet the patients demands	81 (27.4)	123 (41.6)	41 (13.9)	25 (8.4)	26 (8.8)	6 (4)

**Table 7:** Association of median scores with demographic variables.

Variable	Median ABU Score (IQR)	*p*-Value	Median Knowledge Score (IQR)	*p*-Value	Median ABR Mechanism Score (IQR)	*p*-Value	Median FAR Score (IQR)	*p*-Value	Median Attitude Score (IQR)	*p*-Value
Gender *										
Male	1.5 (1.00)	0.235	1.0 (0.88)	0.491	1.5 (0.50)	0.116	1.0 (1.00)	0.527	2.0 (0.50)	0.010
Female	1.0 (1.00)		1.0 (0.50)		1.5 (0.50)		1.0 (1.00)		2.0 (0.50)	
Age (years) **										
19–21	1.5 (1.00)	0.128	1.5 (1.00)	0.001	2.0 (0.50)	0.002	1.0 (1.00)	0.798	2.0 (1.00)	0.043
22 to 24	1.0 (1.00)		1.0 (0.50)		1.5 (1.00)		1.0 (1.00)		2.0 (1.00)	
>24	1.0 (1.00)		1.0 (1.00)		2.0 (0.75)		1.0 (1.25)		2.0 (0.50)	
University type *										
Public	1.5 (1.00)	<0.001	1.5 (1.00)	<0.001	1.5 (1.00)	0.143	1.0 (1.00)	<0.001	2.0 (1.50)	0.002
Private	1.0 (0.88)		1.0 (0.00)		1.5 (1.00)		1.0 (0.00)		2.0 (0.50)	

* Mann–Whitney test, ** Kruskal–Wallis test, ABU = antibiotic use, ABR = antibiotic resistance, FAR = factors affecting antibiotic resistance.

## Data Availability

The dataset used in this study is available from the corresponding author upon reasonable request.

## Supplementary Materials

The following are available online at https://www.mdpi.com/2079-6382/10/1/66/s1, Questionnaire: Understanding of pharmacy students towards antibiotic use, antibiotic resistance and antibiotic stewardship programs: a cross-sectional study from Punjab, Pakistan.
