# Supplementary material for: Understanding of Pharmacy Students towards Antibiotic Use, Antibiotic Resistance and Antibiotic Stewardship Programs: A Cross-Sectional Study from Punjab, Pakistan
Source: Antibiotics (Basel). 2021 Jan 12;10(1):66. doi: 10.3390/antibiotics10010066 (PMC7827071; doi:10.3390/antibiotics10010066)
Supplement: Supplementary file 1 [file antibiotics-10-00066-s001.pdf]

Understanding of Pharmacy Students Towards Antibiotic Use, Antibiotic Resistance and Antibiotic Stewardship Programs: A Cross-Sectional Study from Punjab, Pakistan

Background Information

Antibiotic resistance is the ability of bacteria or other microbes to resist the effects of an antibiotic. Antibiotic resistance occurs when bacteria change in some way that reduces or eliminates the effectiveness of drugs, chemicals, or other agents designed to cure or prevent infections. Antibiotic stewardship is a coordinated program that promotes the appropriate use of antibiotics, improves patient outcomes, reduces microbial resistance, and decreases the spread of infections caused by multidrug-resistant organisms. The Core members of the team include a physician and pharmacist, a clinical microbiologist, infection control personnel, an informatics specialist and a hospital epidemiologist.

Objective

This study aims to assess the understanding of undergraduate pharmacy students towards antibiotic use and antibiotic resistance and antibiotic stewardship programs.

Eligibility criteria

You can participate in this study if you are a student of the final year (fifth year) of your Pharm D (Doctor of Pharmacy) degree program enrolled in Punjab, Pakistan

Consent

Your participation in this study is voluntary. There are no risks involved in this study, and you can decide to withdraw from the data collection at any stage without providing any justification. This is an anonymous survey and does not require your name or email address. If you want to participate in this project, please provide your consent and fill the questionnaire. We assure you that your data will remain confidential and only be used for research purpose.

Contact

If you need to ask anything, please contact Mr Khezar Hayat (khezar.hayat@uvas.edu.pk), Lecturer, Institute of Pharmaceutical Sciences, University of Veterinary and Animal Sciences, Lahore, Pakistan

Part 1: Demographics

1. Age: ___________________ 2. Gender: ☐ Male ☐ Female

3. University: ____

Part 2. Note: Understanding of Phenomenon of Antibiotic Use.

Please Tick in the correct column from options 'yes', 'no', and 'do not know.'

No.	Items	Yes	No	Don't know
**1.**	Antibiotics are the most commonly prescribed anti-infective agents by public health-care sector facilities			
**2.**	Antibiotics are the most commonly prescribed anti-infective agents by private health-care sector facilities			
**3.**	The common cold if treated with antibiotics will make the patients recover more quickly			
**4.**	Antibiotics should be prescribed as preventive measures to fight against future microbial attacks			
**5.**	Antibiotics cannot treat influenza			
**6.**	Antibiotics are indicated to relieve pain			
**7.**	Antibiotics might develop allergy in susceptible individuals			
**8.**	Diphenhydramine is an antibiotic used in treating upper respiratory infections			
**9.**	Cefotaxime belong to the third-generation cephalosporins			
**10.**	Patients can stop taking antibiotics when the symptoms are improving			
**11.**	Keeping the leftover antibiotic course for the next time treatment of the same type of infection is a good practice			
**12.**	Antibiotics treatment can eliminate most of the sensitive bacterial cells from patients			
**13.**	Antibiotics can be obtained without a prescription in Pakistan			
**14.**	Antibiotics are the first line of treatment in sore throat			

(Part 3a) Understanding of Phenomenon of Antibiotic Resistance and antibiotic stewardship program.

Please Tick in the correct column from options 'yes', 'no', and 'do not know.'

No.	Items	Yes	No	Don't know
1	Have you ever heard of antibiotic resistance?			
2	Have you been ever taught antibiotic resistance in your Pharm D Program?			
3	Have you ever heard of antimicrobial stewardship?			
4	Have you been ever taught about antibiotic stewardship in your Pharm D Program?			

(Part 3b) Please Tick in the correct column from options 'yes', 'no', and 'do not know.'

No.	Items	Yes	No	Don't know
1	A resistant bacterium cannot spread in healthcare institutions			
2	Health care workers serve as vectors carrying resistant strains from infected patients to normal patients			
3	Exposure to antibiotics appears to be the principal risk factor for the emergence of antibiotic-resistant bacteria			
4	Inadequate duration of therapy contributes to antibiotic resistance leading to poor patient compliance			
5	Inadequate doses contribute to antibiotic resistance due to poorly designed dosing regimen			
6	Antimicrobial resistance can be minimized through changing empiric therapy to a selected narrow-spectrum therapy in response to the availability of culture and sensitivity results			
7	Cross-resistance is the condition in which resistance occurs to a particular antibiotic that often results in resistance to other antibiotics, usually from a similar class			

**8**	Lack of enforcement regulation sometimes permits antibiotics to be purchased without a prescription from pharmacies			

(Part 4) Understanding of Mechanism of Action of Antibiotic Resistance

Please Tick in the correct column from options 'yes', 'no', and 'do not know.'

No.	Items	Yes	No	Don't know
1,	Beta-lactamase is an enzyme produced by bacteria that breakdown the beta-lactam antibiotics			
2.	Bacteria acquire efflux pumps that extrude the antibacterial agent from the cell before it can reach its target site and exert its effect			
3.	There is no resistance to penicillin for *Streptococcus pyogenes* bacteria			
4.	Bacteriostatic antibiotics are the same as bactericidal antibiotics			
5.	Antibiotic refers to any agent used to kill or inhibit the growth of microorganisms			
6.	*Enterococcus* is a vancomycin-resistant bacterium			

(Part 5) Factors of Antibiotic Resistance.

Please Tick in the correct column from options 'yes', 'no', and 'do not know.'

No	Items	Yes	No	Don't Know
1.	The mutation is a prevalence factor in changing the bacterial protein, which is often the target of antibiotic treatment			
2.	The use of broad-spectrum antibiotics (e.g. 4th generation cephalosporins) as initial therapy for mild infection may increase the risk of antibiotic resistance			
3.	The use of commercially available biocide antiseptics in soaps is highly recommended to patients who have skin infections			

4.	Antibiotic resistance tends to be a feature of urban social change			
5.	Healthcare acquired infections are a breeding ground for antimicrobial resistance			

(Part 6) Attitude Towards Minimizing Antibiotic Resistance

Please Tick in the correct column from five options from strongly agree to strongly disagree.

	**Strongly Agree**	**Agree**	**Neutral**	**Disagree**	**Strongly disagree**
1) Educating health professionals in terms of appropriate antibiotic prescribing reduces the chance of antibiotic resistance	☐1	☐2	☐3	☐4	☐5
2) Formal teaching on proper usage of antimicrobial agents among health care students may minimize the phenomenon of antibiotic resistance	☐1	☐2	☐3	☐4	☐5
3) Antimicrobial education is needed to be well received by healthcare practitioners	☐1	☐2	☐3	☐4	☐5
4) The usage of antibiotics must be related to specialities to enhance the awareness of antibiotic resistance	☐1	☐2	☐3	☐4	☐5
5) Appropriate use of antibiotics may not have any impact on the hospital's total cost expenses on medications	☐1	☐2	☐3	☐4	☐5
6) Prescribing antibiotics over the phone is a good patient care	☐1	☐2	☐3	☐4	☐5
7) The patient should be advised to take part in antibiotic awareness campaigns	☐1	☐2	☐3	☐4	☐5
8) Pharmacists should be encouraged to dispense antibiotics to meet the patients demands	☐1	☐2	☐3	☐4	☐5

Thank you very much for your time taken to complete this questionnaire.
